# Comparison of Reference-Based Assembly and De Novo Assembly for Bacterial Plasmid Reconstruction and AMR Gene Localization in *Salmonella enterica* Serovar Schwarzengrund Isolates

**DOI:** 10.3390/microorganisms10020227

**Published:** 2022-01-20

**Authors:** I-Chen Li, Gine-Ye Yu, Jing-Fang Huang, Zeng-Weng Chen, Chung-Hsi Chou

**Affiliations:** 1 Zoonoses Research Center and School of Veterinary Medicine, National Taiwan University, No. 1, Sec. 4, Roosevelt Rd., Taipei City 106, Taiwan; d06629004@ntu.edu.tw; 2 Animal Technology Research Center, Agricultural Technology Research Institute, No. 52, Kedong 2nd Rd., Zhunan Township, Miaoli County 350, Taiwan; 1082095@mail.atri.org.tw (G.-Y.Y.); hcf@mail.atri.org.tw (J.-F.H.)

**Keywords:** *Salmonella enterica* serovar Schwarzengrund, antimicrobial resistance genes, reference-based assembly, de novo assembly, plasmids

## Abstract

It is well established that plasmids carrying multiple antimicrobial resistance (AMR) genes can be easily transferred among bacterial isolates by horizontal gene transfer. Previous studies have shown that a combination of short- and long-read approaches is effective in reconstructing accurate plasmids. However, high-quality Illumina short reads mapped onto the long reads in the context of an AMR hybrid monitoring strategy have not yet been explored. Hence, this study aimed to improve the reconstruction of plasmids, including the localization of AMR genes, using the above-described parameters on whole-genome sequencing (WGS) results. To the best of our knowledge, this study is the first to use S1 nuclease pulsed-field gel electrophoresis (S1-PFGE) to confirm the number and sizes of plasmids detected by in silico-based predictions in *Salmonella* strains. Our results showed that de novo assembly did not detect the number of bacterial plasmids more accurately than reference-based assembly did. As this new hybrid mapping strategy surpassed de novo assembly in bacterial reconstruction, it was further used to identify the presence and genomic location of AMR genes among three *Salmonella enterica* serovar Schwarzengrund isolates. The AMR genes identified in the bacterial chromosome among the three *Salmonella enterica* serovar Schwarzengrund isolates included: *AAC(3)-IV*, *AAC(6′)-Iy*, *aadA2*, *APH(4)-Ia*, *cmlA1*, *golS*, *mdsA*, *mdsB*, *mdsC*, *mdtK*, *qacH*, *sdiA*, *sul2*, *sul3*, and *TEM*-1 genes. Moreover, the presence of *TEM-1*, *AAC(3)-IV*, *aadA2*, *APH(4)-Ia*, *cmlA1*, *dfrA12*, *floR*, *sul1*, *sul3*, and *tet(A)* genes found within three IncFIB plasmids and one IncX1 plasmid highlight their possible transmission into the environment, which is a public health risk. In conclusion, the generated data using this new hybrid mapping strategy will contribute to the improvement of AMR monitoring and support the risk assessment of AMR dissemination.

## 1. Introduction

The global coronavirus disease (COVID-19) pandemic, caused by the severe acute respiratory syndrome coronavirus 2 (SARS-CoV-2), has infected more than 245 million people and has caused over 4 million deaths worldwide since its first identification in 2019 [1]. While the world is collaborating to stop the spread of COVID-19, it is equally important to be prepared for the direct and indirect impacts of this pandemic on the development of antimicrobial resistance (AMR). In fact, in addition to the direct cytotoxic effects of this deadly virus, bacterial co-infections and secondary infections have become prevalent and emergent, especially among patients admitted to intensive care units due to COVID-19 [2]. Studies have shown that after viral infections, such as influenza or COVID-19, both the innate and adaptive immune systems are impaired and thereby enhance the proliferation of potentially pathogenic bacterial species [3]. Recently, a study conducted by The Pew Charitable Trusts found that among 5838 hospital admissions from February through July 2020, more than half of hospital patients received antibiotics during the first six months of the pandemic to prevent secondary bacterial and fungal infections [4]. However, in 96% of cases, treatment was prescribed before a bacterial infection was even confirmed [4]. Moreover, along with physicians overprescribing antibiotics as part of defensive medicine, increased hygiene procedures have been introduced, including hand sanitizers, surface disinfectants, and personal protective equipment, during the pandemic era [5]. Ultimately, these factors may cause increased rates of antimicrobial resistance in pathogenic microbes and present a significant global health threat in the coming years.

Currently, approximately 2.8 million people worldwide are infected with antibiotic-resistant bacteria and approximately 700,000 die per year around the globe as a result of antibiotic-resistant infections [6]. Bacteria can acquire AMR through microbial chromosomal mutation or the acquisition of mobile genetic elements, such as plasmids [7]. Each bacterium can hold one or multiple plasmids with varying sizes and copy numbers, which can spread horizontally among isolates and across the species or phyla barriers, promoting the evolution of resistance [8]. To combat the increasing threat of AMR, the use of whole-genome sequencing (WGS) has proven useful for investigating infectious disease pathogen transmission, and its data can now be obtained at a relatively low cost in a fairly short period of time [9]. However, most WGS-based studies have often focused on the chromosome of a resistant host strain instead of circulating plasmids [10]. One of the biggest reasons for this is that plasmids often contain repeated sequences that are shared with the chromosomal genome and are therefore difficult to construct using short-read data [11].

The introduction of third-generation sequencers (MinION nanopore; Oxford Nanopore Technologies) may afford one solution to this challenge. Compared to second-generation sequencing platforms that generate high throughput and shorter read lengths (150–300 bp), MinION offers longer reads (>10 kb) that can capture entire genomes including extraneous elements [12]. Although long reads can resolve repeat elements in bacterial genomes and improve the assembly contiguity, their drawbacks include a significantly higher error rate that introduces single-base substitutions and short insertions/deletions in genome assemblies [13]. Since both second- and third-generation sequencing have their limitations, a combination of short-and long-read sequence data seems to be a promising strategy to resolve complete plasmid sequences from whole bacterial genome sequencing. In fact, previous studies have shown that this kind of hybrid assembly approach is effective in reconstructing accurate plasmids containing AMR genes in bacterial pathogens [14,15]. However, a hybrid strategy using high-quality Illumina short reads mapped onto the long reads in the context of AMR monitoring has not yet been explored. Since plasmids play a key role in disseminating AMR genes, it is imperative to capture and sequence these mobile elements in order to improve risk assessment. Therefore, this study is the first to improve the reconstruction of plasmids, including the localization of AMR genes, using the above-described parameters on WGS results.

According to the latest data collected by the European Center for Disease Prevention and Control (ECDC) and the European Food Safety Authority (EFSA), a large proportion of *Salmonella* bacteria from humans, animals, and food are multidrug-resistant, and infections with foodborne bacteria are thus becoming harder to treat [16]. As a follow-up study, this study selected three multidrug-resistant *Salmonella enterica* serovar Schwarzengrund strains with multiple plasmids isolated from animal sources [17,18], which made them ideal strains to reconstruct plasmids. The reconstruction of plasmids was first compared using data from a reference-based assembly of Illumina short reads mapped onto the long reads and a de novo assembly of both short and long reads. The results obtained by these two approaches were compared to those of the S1 nuclease pulsed-field gel electrophoresis (S1-PFGE) analysis, which verified the number and sizes of the plasmids present in these three strains. Finally, we compared the results of each technology to assess their capacity for detecting antimicrobial resistance genes.

## 2. Materials and Methods

### 2.1. Bacterial Isolates and DNA Preparation

Three multidrug-resistant *Salmonella enterica* serovar Schwarzengrund strains (SS09, SS12, and SS15) with multiple plasmids isolated from animal sources were prepared according to our previous studies [17,18] (Table S1). In brief, these strains were revived in tryptic soy broth and grown overnight at 37 °C. Then, genomic DNA from each strain isolated from the overnight culture was extracted using a DNeasy Blood and Tissue Kit (Qiagen, Valencia, CA, USA) following the manufacturer’s instructions.

### 2.2. Whole-Genome Sequencing

**Figure 1:**
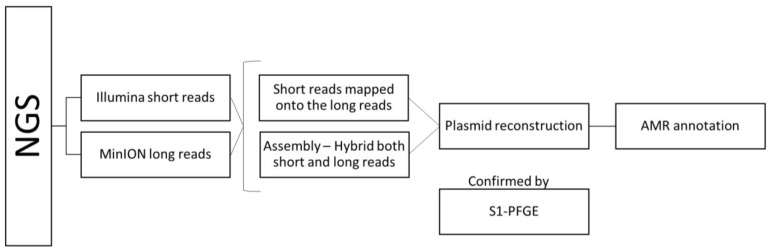
Overview of workflow to reconstruct plasmids and localize antimicrobial resistance genes in *Salmonella enterica* serovar Schwarzengrund isolates.

The workflow used in this study is visualized in Figure 1.

For Illumina short-read sequencing, 1.5 µg DNA was used for library preparation employing the TruSeq DNA preparation kit (Illumina, San Diego, CA, USA) and sequenced on a NextSeq500 instrument (Illumina, Inc., San Diego, CA, USA) with the 150PE protocol according to the manufacturer’s instructions. Trimming of the short reads was performed with the Trimmomatic software (version 0.36) developed by Bolger et al. [19]. The filtered data with an average Phred quality score ≥Q20 and read length ≥30 bp were used for subsequent mapping or genome assemblies. The short raw reads were deposited in the NCBI database under BioProject # PRJNA635494.

For MinION long-read sequencing, 1.5 µg DNA was used for library preparation employing the 1D ligation-based sequencing kit (SQK-LSK-109, Oxford Nanopore, Littlemore, UK) and Native Barcoding Expansion 1-12 (EXP-NBD104, Oxford Nanopore, Littlemore, UK), which was then sequenced with a FLO-MINSP6 R9.4.1 flow cell coupled to a MinION Mk1B sequencer (Oxford Nanopore Technologies, Oxford, Littlemore, UK) connected to a MinKNOW GUI version 4.3.20 device (including Guppy base caller version 5.0.11) as recommended by the manufacturer. Base-calling of these sequences was performed with the Guppy software (version 4.2.2) developed by Oxford Nanopore Technologies [20]. The trimming of the adaptor was performed with the Porechop software (version 0.2.4) developed by Wick R [21]. Data used for subsequent mapping or genome assemblies were filtered with NanoFilt (version 2.8.0) [22] for having an average Phred quality score ≥Q7 and read length ≥1000 bp. The long raw reads were deposited in the NCBI database under BioProject # PRJNA635494.

To calculate the coverage of the genome, the Lander–Waterman equation [23] was used as follows:

(1) C=LN/G

where *C* is the coverage estimated by read mapping, *L* is the read length, *N* is the total number of reads, and *G* is the haploid genome length.

### 2.3. De Novo Assembly of Both Short and Long Reads

Before de novo assembly, the quality of the short reads and long reads was checked with FastQC (version 0.11.9) [24] and LongQC (version 1.2) [25], respectively. Then, the de novo assembly of both the short and long reads was generated with Unicycler (version 0.4.8) [26] under default parameters (normal mode). The assembly statistics were then retrieved with QUAST (version 5.0.2) [27].

### 2.4. Reference-Based Assembly of Illumina Short Reads Mapped onto the Long Reads Assembly

The assembly of long reads was first carried out using Unicycler with the default parameters (normal mode). Mapping the short reads to the long-read assembly was performed using BWA-MEM (version 2.2.1) [28] with the “-t 4, -R, and -M” parameters, and the resulting sequence alignment was sorted and indexed using SAMtools (version 1.11) with the default settings [29]. The detection of indels (1–50 bp) was carried out using GATK (version 3.8) [30]. Finally, a consensus sequence for each isolate was generated using Bcftools (version 1.14) under the default settings [31] and SeqTk (version 1.3) with the “-aQ64 -q30 -n N -U -A” parameters [32]. Mapping evaluations were then performed using QUAST (version 5.0.2).

### 2.5. Plasmid Annotation

Genomes from both the reference-based assembly and de novo assembly were screened for known plasmid genes using ABRicate (version 0.8.13) [33] against the PlasmidFinder database [34]. A plasmid sequence identity of 85% and a coverage of 80% were used as thresholds as recommended by the Food and Drug Administration (FDA) [35].

### 2.6. S1-PFGE

The numbers and sizes of plasmids were estimated by S1-PFGE analysis as described previously [36]. *S. enterica* serovar Braenderup H9812 was used for size determination. In brief, the genomic DNA was prepared in agarose blocks, digested with 10 units of P1 nuclease (M0660S, New England Biolabs, Ipswich, MA, USA), and then separated on a 1% certified megabase agarose gel in 0.5× Tris-borate-EDTA buffer at 14 °C for 20.5 h with pulse times between 2.16 and 63.8 s using a CHEF-Mapper XA PFGE system (Bio-Rad, Hercules, CA, USA). Plasmids less than 10 kb in size were further analyzed by running them on a 1.2% agarose gel in 0.5× Tris-borate-EDTA buffer at 120 V for 30 min with a Bio-1Kb Mass DNA Ladder (Protech Technology, Taipei, Taiwan) used as a marker. The gels were then stained with ethidium bromide, visualized under UV light, and photographed. The fingerprints were analyzed using BioNumerics (version 7.1).

### 2.7. Antimicrobial Resistance Gene Identification and Location

AMR genes were identified by screening the assembled sequences (from both reference-based assembly and de novo assembly) with ABRicate against CARD databases [37]. The location of AMR is based on its placement within the contigs from genome assemblies.

## 3. Results

### 3.1. Quality Control of Short and Long Reads

**Figure 2:**
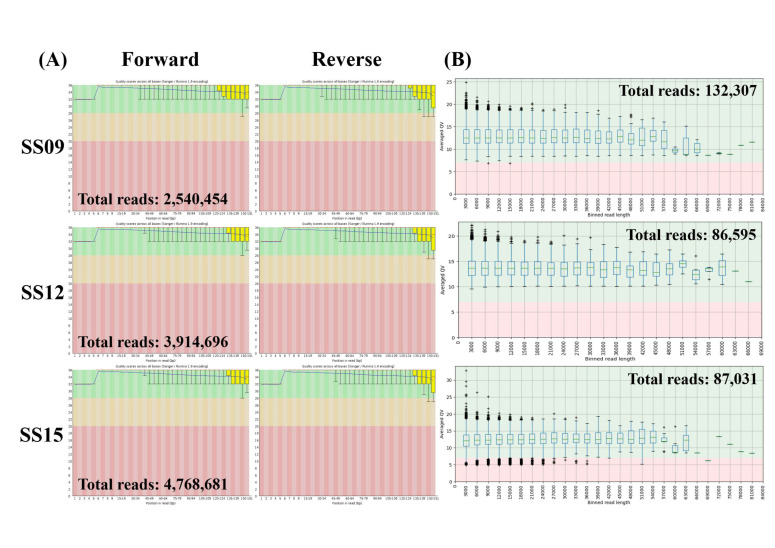
(**A**) FastQC and (**B**) LongQC quality assessment of three *Salmonella enterica* serovar Schwarzengrund isolates (SS09, SS12, and SS15) after trimming and adapter removal.

After trimming and adapter removal, the quality of the short reads (Figure 2A) and long reads (Figure 2B) of three *S. enterica* serovar Schwarzengrund isolates were assessed by FastQC and LongQC, respectively. All samples passed the mean quality score test in FastQC (average Phred score of 20) and LongQC (average QV score of 7) (Figure 2). The total short (mean length of 88 bp) and long reads (mean length of 7131 bp) for SS09, SS12, and SS15 were 2,540,454, 3,914,696, 4,768,681, and 132,307, 86,565, 87,031, respectively. Assuming that the size of the *S. enterica* serovar Schwarzengrund genome is ~4.8 Mb, this represents a total coverage of the genome range from 46 to 87× for the short-reads and from 128 to 196× for the long-reads.

### 3.2. Comparison of De Novo and Reference-Based Assembly

**Figure 3:**
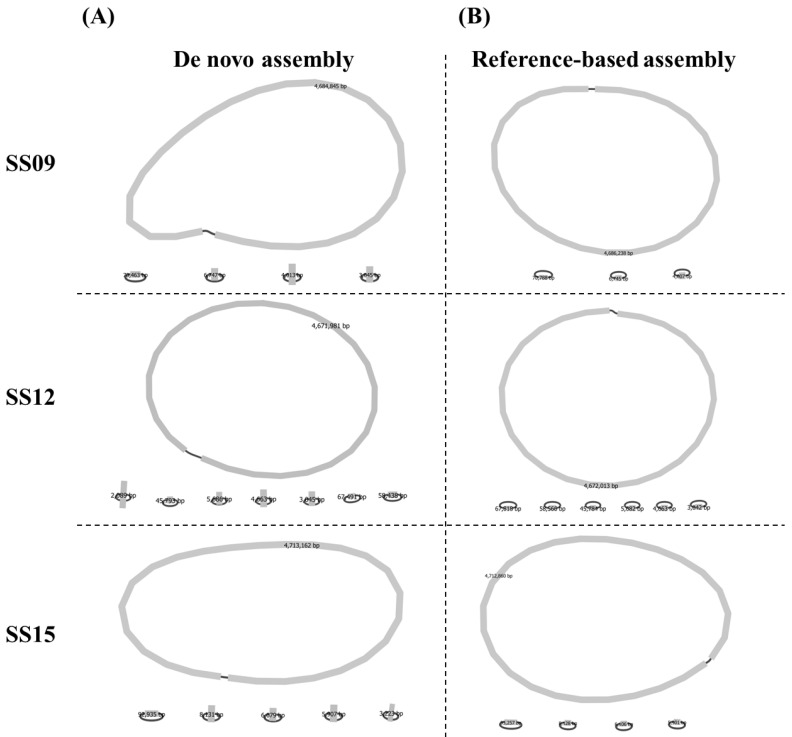
Bandage visualizations of three *Salmonella enterica* serovar Schwarzengrund isolates (SS09, SS12, and SS15) using (**A**) de novo and (**B**) reference-based assembly approaches.

After the quality of the Illumina short reads and long reads was confirmed, reference-based assembly and de novo assembly approaches were then performed to construct complete chromosomes and plasmids in the three *S. enterica* serovar Schwarzengrund isolates, SS09, SS12, and SS15 (Figure 3). The assembly graphs were first visualized with Bandage and all isolates reported one circular chromosomal contig range from 4,767,778 to 4,859,186 bp. The results for GC content in the three isolates were also similar despite the two different assembly methods used. However, multiple plasmid contigs of varying sizes were found by the two different assembly approaches. Based on the obtained statistics calculated using QUAST (Table 1), there were four (with sizes of 70,463, 6747, 4013, and 3045 bp), seven (with sizes of 67,491, 58,438, 45,793, 5686, 4663, 3045, and 2089 bp) and five (with sizes of 92,935, 8131, 6079, 5907, and 3223 bp) plasmid contigs for SS09, SS12, and SS15, respectively, found using the de novo assembly approach. In comparison, reference-based assembly only produced three (with sizes of 70,788, 6745, and 4007 bp), six (with sizes of 67,818, 58,566, 45,784, 5682, 4663, and 3042 bp), and four (with sizes of 93,257, 8128, 6106, and 5901 bp) plasmid contigs for SS09, SS12, and SS15, respectively.

### 3.3. Plasmid Annotation by PlasmidFinder and Confirmation by S1-PFGE Analysis

**Figure 4:**
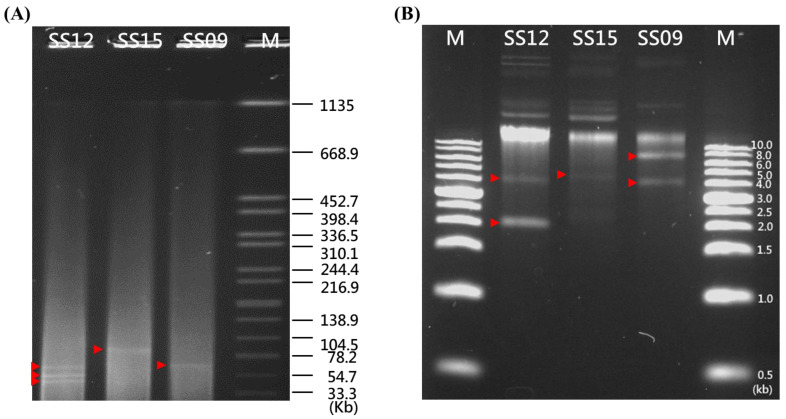
S1 nuclease pulsed-field gel electrophoresis (S1-PFGE) gel images showing the presence (labeled by red arrows) of (**A**) large (>30 kb) and (**B**) small (<10 kb) plasmids in three *Salmonella enterica* serovar Schwarzengrund isolates (SS09, SS12, and SS15). For large plasmids, *S. enterica* serovar Braenderup H9812 was used as the marker, while for small plasmids, Bio-1Kb Mass DNA Ladder was used as the marker. Plasmids are indicated by red arrows.

The circularized plasmid contigs from each sample identified in silico (Table 1 and Table 2) and the number and size of the plasmids detected by the S1-PFGE and agarose gel analyses (Figure 4A,B, respectively) were then compared to obtain an overview of the mobile genetic elements. A total of three (with sizes of approximately 70 kb, 7 kb, and 4 kb), five (with sizes of approximately 67 kb, 58 kb, 45 kb, 4 kb, and 2 kb), and two (with sizes of approximately 93 kb and 5 kb) plasmid bands were identified in the SS09, SS12, and SS15 strains, respectively, by the S1-PFGE and agarose gel analyses (Figure 4A,B, respectively), which matched more closely with the estimated number of plasmid contigs from the reference-based assembly approach and not the de novo assembly approach (Table 1). Since S1-PFGE and the agarose gel showed no evidence of their biological presence, the de novo assembly approach mistakenly recognized a 3 kb plasmid in SS09 isolates; two plasmids of 5 and 3 kb in SS12 isolates; and three plasmids of 8, 5, and 3 kb in SS15 isolates, while the reference-based assembly only mistakenly recognized two plasmids of 5 and 3 kb in SS12 isolates and two plasmids of 8 and 5 kb in SS15 isolates. Moreover, except for the de novo assembly method that identified a 2089 bp Col(BS512) plasmid within the SS12 isolate, both the de novo and reference-based assembly approaches recognized one large plasmid (70 kb IncFIB(K)) and two small plasmids (7 kb Col156 and 4 kb Col440II) within the *S. enterica* serovar Schwarzengrund SS09 isolate, three large plasmids (67 kb IncFIB(K), 58 kb IncL/M, and 45 kb IncX1) and one small plasmid (4 kb Col156) within the SS12 isolate, and one large plasmid (93 kb IncFIB(K)) and one small plasmid (6 kb ColRNAI) within the SS15 isolate (Table 2).

### 3.4. Antimicrobial Resistance Gene Identification and Location

To obtain the full picture of the AMR gene content, a search on the de novo and reference-based assemblies using the CARD tool was performed (Table 3). Both the de novo and reference-based assembly approaches achieved similar results. For the SS09 isolate, the chromosome (contig 1) carried the AMR genes *AAC(3)-IV*, *AAC(6′)-Iy*, *aadA2*, *APH(4)-Ia*, *cmlA1*, *golS*, *mdsA*, *mdsB*, *mdsC*, *mdtK*, *qacH*, *sdiA*, *sul3*, and *TEM-1*, while its IncFIB(K) plasmid (contig 2) contained AMR *aadA2*, *dfrA12*, *sul1*, and *tet(A)* genes. For the SS12 isolate, the chromosome (contig 1) carried the AMR genes *mdtK*, *sdiA*, *AAC(6′)-Iy*, *TEM-1*, *aadA2*, *cmlA1*, *qacH*, *sul2*, *golS*, *mdsA*, *mdsB*, and *mdsC* while its IncFIB(K) plasmid (contig 2) and its IncX1 plasmid (contig 4) contained AMR *tet(A)*, *dfrA12*, and *aadA2*, and *TEM-1*, *AAC(3)-IV*, *APH(4)-Ia*, and *floR* genes, respectively. For the SS15 isolate, the chromosome (contig 1) carried the AMR genes *AAC(6′)-Iy*, *golS*, *mdsA*, *mdsB*, *mdsC*, *mdtK*, and *sdiA*, while its IncFIB(K) plasmid (contig 2) contained AMR *AAC(3)-IV*, *aadA2*, *APH(4)-Ia*, *cmlA1*, *dfrA12*, *floR*, *qacH*, *sul1*, *sul3*, and *tet(A)* genes.

## 4. Discussion

Multidrug-resistant *Salmonella enterica* bacteria constitutes a significant public health concern due to the potential transmission to humans at the end of the food chain. As AMR genes localized on plasmids can be easily transferred and spread by horizontal gene transfer, their occurrence should be closely monitored. Hence, in the current study, a strategy to reconstruct bacterial plasmids and fully characterize the presence of AMR genes and their genomic location in *S. enterica* serovar Schwarzengrund isolates was developed. Moreover, as a follow-up study, three multidrug-resistant *S. enterica* serovar Schwarzengrund isolates with multiple plasmids of different size ranges were chosen for in-depth characterization [17,18].

In NGS data, sequencing is a critical step as it can affect the downstream analysis and interpretation processes. As this study used two assembly approaches, including a reference-based assembly of Illumina short reads mapped onto the long reads and a de novo assembly of both short and long reads, to reconstruct chromosome and plasmids, the quality of the short and long reads was first examined. Overall, after trimming and adapter removal, all sequencing reads met the recommended requirements from Illumina and Oxford Nanopore for accuracy, coverage, read length, and read counts [38]. Moreover, consistent with a previous publication that produced a genome coverage of 50–150× for short reads and 59–250× for long reads in multidrug-resistant *Salmonella enterica* strains [39], this study generated a 46 to 87× coverage of the Illumina short sequencing data in combination with a 128 to 196× coverage of the Oxford Nanopore long sequencing data, suggesting the similar use of high-quality sequencing runs to produce accurate genome assemblies.

Next, the reference-based assembly and de novo assembly approaches were assessed for their suitability for bacterial chromosome and plasmid reconstruction. In all isolates, both methods allowed the closing of the chromosomal contig. However, for plasmid reconstruction, the de novo assembly approach appeared to detect one additional plasmid in all isolates when compared to those identified by the reference-based assembly approach. Under the assumption that all the plasmids were detected by S1-PFGE, de novo assembly could identify a 2089 bp Col(BS512) plasmid within the SS12 isolate, while both methods had plasmids that were not recognized by the S1-PFGE and agarose gel analyses, such as the 3 kb plasmid in SS09 isolates; two plasmids of 5 and 3 kb in the SS12 isolates; and three plasmids of 8, 5, and 3 kb in the SS15 isolates identified by the de novo assembly approach, and the two plasmids of 5 and 3 kb in the SS12 isolates and two plasmids of 8 and 5 kb in the SS15 isolates detected by the reference-based assembly. According to the previous study [40], the assemblies sometimes resulted in the detection of plasmids that were not detectable by S1-PFGE and agarose gel analyses. This could be due to the long-read assemblies often being error-prone and containing a higher percentage of duplicated genes [41], potentially leading to misinterpretations. Moreover, as long-read sequencing has size-selection and bead clean-up steps that could exclude short extrachromosomal DNA elements [42], this may also lead to the exclusion of small plasmids, such as the 2089 bp Col(BS512) plasmid found within the SS12 isolate. Nevertheless, de novo assembly could not detect the number more accurately than reference-based assembly, probably due to the misalignment of short reads [43]; this suggests the superiority of the reference-based assembly approach for plasmid reconstruction of a size range greater than 2 kb.

According to a previous study, plasmids of sizes greater than 80 kb were detected when S1-PFGE was used as a method to screen for the presence of plasmids in *Salmonella indiana* isolates [44]. In this study, S1-PFGE was able to detect plasmids of sizes greater than 33.3 kb, which confirmed their presence by in silico-based prediction, suggesting that S1-PFGE may be efficient in screening for large plasmids. In fact, S1-PFGE is regarded as a good method for the screening of mega plasmids with sizes above 100 kb [36]. However, PFGE is not efficient in screening for small plasmids. According to a previous study, the alkaline lysis method detected 89 plasmids of sizes 0–45 kb in *Campylobacter*, while the PFGE method only detected 20 plasmids of sizes 0–45 kb [45]. In another study, the alkaline lysis method detected 542 plasmids smaller than 90 kb among 222 *Staphylococcus aureus* isolates, while PFGE identified only 151 plasmids within that size range [46]. As this study used S1-PFGE and not the alkaline lysis method for plasmid isolation, this likely explains the inconsistency in the number of small plasmids less than 33.3 kb detected between PFGE and in silico-based prediction. In future studies, to confirm the presence of small plasmids less than 33.3 kb, different plasmid isolation methods should be tested.

As shown in previous studies, complete plasmid assembly is frequently impossible using short-read sequencing [11]; therefore, it was unknown whether the AMR genes were localized on the bacterial chromosome or plasmids. The correct determination of AMR gene location is of high importance, as a chromosomal AMR gene can only spread clonally within a population; however, if the AMR genes are localized on a plasmid, they can spread to other bacterial pathogens [47]. As this study accurately reconstructed the plasmids, it was possible to determine the exact location of the AMR genes on the chromosome and plasmid. The results of this study showed that all assemblies resulted in the same AMR gene prediction and location for all three strains. Moreover, for the first time, the AMR genes identified in the bacterial chromosome among three *Salmonella enterica* serovar Schwarzengrund isolates included *AAC(3)-IV*, *AAC(6′)-Iy*, *aadA2*, *APH(4)-Ia*, *cmlA1*, *golS*, *mdsA*, *mdsB*, *mdsC*, *mdtK*, *qacH*, *sdiA*, *sul2*, *sul3*, and *TEM*-1, which encode resistance to aminoglycosides (*AAC(3)-IV*, *AAC(6′)-Iy*, *aadA2*, *APH(4)-Ia*), chloramphenicol (*cmlA1*), multiple drugs (*golS*, *mdsA*, *mdsB*, *mdsC*, *mdtK*, *qacH*, *sdiA*), sulfonamides (*sul2*, *sul3*), and ampicillin (*TEM*-1).

Nevertheless, the presence of three IncFIB plasmids and one IncX1 plasmid found among the isolates that carried several AMR genes was particularly worrisome, as they increase the risk of spreading multiple resistance genes. Although the AMR genes (*TEM-1*, *AAC(3)-IV*, *APH(4)-Ia*, and *floR*) carried by the IncX1 plasmid in this study were completely different from those reported in previous work (*repA*, *qnrS1*, *tet(A)*, *cmlA1*, *sul3*, and *bla_TEM-1B_* genes) [48,49,50], the current observations could be well-explained by prior research showing that isolates originating from different countries belonging to different lineages may be exposed to different environments and therefore carry different resistance genes [51]. On the other hand, all isolates carried an IncFIB plasmid that could increase colonization in the chicken cecum, which may help to explain its persistence in the food animal population [52]. Within the plasmid IncFIB, some of the common resistance genes identified were similar to those previously reported [53], including *AAC(3)-IV*, *aadA2*, *APH(4)-Ia*, *cmlA1*, *dfrA12*, *floR*, *sul1*, *sul3,* and *tet(A)*, which encode resistance to gentamicin, streptomycin, hygromycin B, chloramphenicol, trimethoprim, florphenicol, sulfonamides, and tetracycline, respectively. It should be noted that gentamicin is a critically important antimicrobial, while chloramphenicol, trimethoprim, sulfonamides, and tetracycline are highly important antibiotics in human medicine, according to the latest WHO publication [54]. By using this new hybrid mapping strategy, we were able to determine the exact location of AMR genes, which highlights a new control and prevention strategy to combat the increasing threat of AMR.

This study compared two different approaches involved in the accurate reconstruction of the bacterial chromosome and plasmids as well as the determination of the exact location of AMR genes. As the reference-based assembly surpassed de novo assembly for bacterial plasmid reconstruction, this new hybrid mapping strategy further identified the presence and genomic location of AMR genes among *Salmonella enterica* serovar Schwarzengrund isolates that have been ignored in the past.

## Figures and Tables

**Table 1:** Statistics of de novo and reference-based assembly results for three *Salmonella enterica* serovar Schwarzengrund isolates (SS09, SS12, and SS15) calculated using QUAST.

	De Novo Assembly			Reference-Based Assembly		
Strains	SS09	SS12	SS15	SS09	SS12	SS15
Total length (bp)	4,769,113	4,859,186	4,829,437	4,767,778	4,857,568	4,826,252
GC (%)	52.19	52.13	52.21	51.19	52.14	52.21
N50	4,684,845	4,671,981	4,713,162	4,686,238	4,672,103	4,712,860
L50	1	1	1	1	1	1
Contig	5	8	6	3	7	5
Size contig 1 (bp)	4,684,845	4,671,981	4,713,162	4,686,238	4,672,198	4,712,860
Size contig 2 (bp)	70,463	67,491	92,935	70,788	67,818	93,257
Size contig 3 (bp)	6747	58,438	8131	6745	58,566	8128
Size contig 4 (bp)	4013	45,793	6079	4007	45,784	6106
Size contig 5 (bp)	3045	5686	5907		5682	5901
Size contig 6 (bp)		4663	3223		4663	
Size contig 7 (bp)		3045			3042	
Size contig 8 (bp)		2089				

**Table 2:** Plasmid genes detected with PlasmidFinder by de novo and reference-based assembly approaches in three *Salmonella enterica* serovar Schwarzengrund isolates (SS09, SS12, and SS15).

De Novo Assembly				Reference-Based Assembly			
Replicon	Contig	Identity (%)	Coverage (%)	Replicon	Contig	Identity (%)	Coverage (%)
	SS09				SS09		
IncFIB(K)	2	98.93	100	IncFIB(K)	2	98.93	100
Col156	3	98.03	98.7	Col156	3	98.03	98.7
Col440II	4	96.44	99.65	Col440II	4	96.44	99.65
	SS12				SS12		
IncFIB(K)	2	98.93	100	IncFIB(K)	2	98.75	99.82
IncL/M	3	94.63	89.46	IncL/M	3	94.46	89.31
IncX1	4	98.93	100	IncX1	4	98.93	100
ColRNAI	5	90.84	100	ColRNAI	5	90.08	99.23
Col156	6	93.51	100	Col156	6	93.51	100
Col(BS512)	8	100	100				
	SS15				SS15		
IncFIB(K)	2	98.93	100	IncFIB(K)	2	98.93	100
IncQ1	3	100	81.28	IncQ1	3	100	81.28
ColRNAI	4	87.79	100	ColRNAI	4	83.97	99.23

**Table 3:** Antimicrobial resistance genes detected with CARD from de novo and reference-based assembly approaches in three *Salmonella enterica* serovar Schwarzengrund isolates (SS09, SS12, and SS15).

De Novo Assembly				Reference-Based Assembly			
Resistance Gene	Contig	Identity (%)	Coverage (%)	Resistance Gene	Contig	Identity (%)	Coverage (%)
	SS09				SS09		
*AAC(3)-IV*	1	100	100	*AAC(3)-IV*	1	100	100
*AAC(6′)-Iy*	1	98.4	100	*AAC(6′)-Iy*	1	98.4	100
*aadA2*	1	100	100	*aadA2*	1	100	100
*aadA2*	2	100	100	*aadA2*	2	99.87	100
*APH(4)-Ia*	1	100	100	*APH(4)-Ia*	1	100	100
*cmlA1*	1	99.92	100	*cmlA1*	1	99.92	100
*dfrA12*	2	100	100	*dfrA12*	2	100	100
*golS*	1	99.36	100	*golS*	1	99.36	100
*mdsA*	1	98.78	100	*mdsA*	1	98.78	100
*mdsB*	1	99.02	100	*mdsB*	1	99.02	100
*mdsC*	1	98.28	100	*mdsC*	1	98.28	100
*mdtK*	1	98.88	100	*mdtK*	1	98.88	100
*qacH*	1	91.59	100	*qacH*	1	91.59	100
*sdiA*	1	98.75	100	*sdiA*	1	98.75	100
*sul1*	2	100	100	*sul1*	2	99.88	99.88
*sul3*	1	100	100	*sul3*	1	100	100
*TEM-1*	1	99.88	100	*TEM-1*	1	99.88	100
*tet(A)*	2	100	97.8	*tet(A)*	2	99.68	97.65
	SS12				SS12		
*AAC(3)-IV*	4	100	100	*AAC(3)-IV*	4	99.87	99.87
*AAC(6′)-Iy*	1	98.4	100	*AAC(6′)-Iy*	1	98.4	100
*aadA2*	1	100	100	*aadA2*	1	100	100
*aadA2*	2	100	100	*aadA2*	2	99.87	100
*APH(4)-Ia*	4	100	100	*APH(4)-Ia*	4	100	100
*cmlA1*	1	99.92	100	*cmlA1*	1	99.92	100
*dfrA12*	2	100	100	*dfrA12*	2	100	100
*floR*	4	99.75	100	*floR*	4	99.75	100
*golS*	1	99.36	100	*golS*	1	99.36	100
*mdsA*	1	98.78	100	*mdsA*	1	98.78	100
*mdsB*	1	99.02	100	*mdsB*	1	99.02	100
*mdsC*	1	98.28	100	*mdsC*	1	98.28	100
*mdtK*	1	98.88	100	*mdtK*	1	98.88	100
*qacH*	1	91.59	100	*qacH*	1	91.59	100
*sdiA*	1	98.75	100	*sdiA*	1	98.75	100
*sul2*	1	100	100	*sul2*	1	99.88	99.88
*TEM-1*	1	99.88	100	*TEM-1*	1	99.88	100
*TEM-1*	4	99.88	100	*TEM-1*	4	99.88	100
*tet(A)*	2	100	97.8	*tet(A)*	2	100	97.8
	SS15				SS15		
*AAC(3)-IV*	2	100	100	*AAC(3)-IV*	2	100	100
*AAC(6′)-Iy*	1	98.4	100	*AAC(6′)-Iy*	1	98.4	100
*aadA2*	2	99.87	100	*aadA2*	2	99.87	100
*APH(4)-Ia*	2	100	100	*APH(4)-Ia*	2	100	100
*cmlA1*	2	99.92	100	*cmlA1*	2	99.92	100
*dfrA12*	2	100	100	*dfrA12*	2	100	100
*floR*	2	99.67	100	*floR*	2	99.59	99.92
*golS*	1	99.36	100	*golS*	1	99.36	100
*mdsA*	1	98.78	100	*mdsA*	1	98.78	100
*mdsB*	1	99.02	100	*mdsB*	1	98.83	99.81
*mdsC*	1	98.21	100	*mdsC*	1	98.21	100
*mdtK*	1	98.88	100	*mdtK*	1	98.88	100
*qacH*	2	91.59	100	*qacH*	2	91.59	100
*sdiA*	1	98.75	100	*sdiA*	1	98.75	100
*sul1*	2	100	100	*sul1*	2	100	100
*sul3*	2	100	100	*sul3*	2	99.87	99.87
*tet(A)*	2	100	97.8	*tet(A)*	2	99.84	97.65

## Data Availability

The short and long raw reads used in this study were deposited to the NCBI database under BioProject accession number PRJNA635494.

## Supplementary Materials

The following supporting information can be downloaded at: www.mdpi.com/article/10.3390/microorganisms10020227/s1, Table S1: Three multidrug-resistant *Salmonella enterica* serovar Schwarzengrund strains used in this study.
