# Supplementary material for: Comparison of Reference-Based Assembly and De Novo Assembly for Bacterial Plasmid Reconstruction and AMR Gene Localization in Salmonella enterica Serovar Schwarzengrund Isolates
Source: Microorganisms. 2022 Jan 20;10(2):227. doi: 10.3390/microorganisms10020227 (PMC8874696; doi:10.3390/microorganisms10020227)
Supplement: Supplementary file 1 [file microorganisms-10-00227-s001.zip › microorganisms-1552373-supplementary.pdf]

Table S1. Three multidrug-resistant *Salmonella enterica* serovar Schwarzengrund strains used in this study

Sample ID	Sources	Isolation Year
SS09	Broiler	2008
SS12	Broiler	2010
SS15	Turkey	2012
